# A Novel Strategy to Eliminate the Influence of Water Adsorption on Quartz Surfaces on Piezoelectric Dynamometers

**DOI:** 10.3390/s16071060

**Published:** 2016-07-08

**Authors:** Zhenyuan Jia, Lei Jin, Wei Liu, Zongjin Ren

**Affiliations:** 1 Key Laboratory for Precision and Non-traditional Machining Technology of the Ministry of Education, Dalian University of Technology, Dalian 116024, China; jzyxy@dlut.edu.cn (Z.J.); dlutjl@aliyun.com (L.J.); Renzongjin@dlut.edu.cn (Z.R.); 2 Liaoning Provincial College of Communications, Shenyang 110122, China

**Keywords:** piezoelectric quartz crystals, adsorption of water, sheet resistance, roughness, fluorination

## Abstract

Piezoelectric dynamometers are out of use in high humidity. Experimental results showed that piezoelectric coefficients measured by the force-induced charges method initially fluctuated in a small range and then was unstable, and they could not be measured at high relative humidity (RH). The traditional shielding method-insulation paste was not quiet convenient, and it even added the weight of piezoelectric dynamometers. In this paper, a novel strategy that eliminates the influence of water adsorption with quartz surfaces on piezoelectric dynamometers was proposed. First, a water-quartz model was developed to analyze the origin of the RH effect. In the model, water vapor, which was adsorbed by the quartz sheet side surface, was considered. Second, equivalent sheet resistor of the side surface was researched, while the relationship of the three R’s (Roughness, RH, and Resistor) was respectively discussed based on the adsorption mechanism. Finally, fluorination technology was skillfully adapted to each surface of quartz sheets to shield the water vapor. The experiment verified the fluorination strategy and made piezoelectric dynamometers work in high humidity up to 90%RH successfully. The results showed that the presented model above was reasonable. In addition, these observations also drew some useful insights to change the structure of piezoelectric dynamometers and improve the properties.

## 1. Introduction

Water is the most common material in nature. As the high surface energy of solid substance, ubiquitous vapor has the propensity to be adsorbed onto them. At the solid-water interface, adsorbed water not only gives rise to a multitude of specific behaviors but also immensely changes the properties of both substances, so it plays an essential role in gas-sensing sensors [1], relative humidity sensors [2,3], and hydrophobic and hydrophilic surfaces [4,5].

Silicon oxide is a significant material and extensively used. Meanwhile, the silica–water interface is extensively researched. Notman and Walsh [6] characterized the interaction of water with fully hydroxylated α-quartz for peptides binding to quartz. The result showed that two layers of ordered water on the quartz surfaces were found and the (1 01 0) surface was hydrophobic moieties. Moreover, β-quartz adsorption with water was studied in Bonnaud’s [7] paper. Miranda [8] researched water monolayer adsorbed on mica. Around 90% relative humidity (RH), the water coverage reached a full monolayer and an ice-like structure appeared. David and Seong [9] elucidated the relative-humidity dependence of the adhesion force of nano-asperity contacted on silicon oxide surfaces. An ice-bridge rupture should be taken into account to explain the magnitude and shape dependence. Therefore, different kinds of physical forms of silica were researched from two main subjects: the mechanism and structure of adsorption. However, piezoelectric quartz was still lacking.

Piezoelectric quartz crystals [10], based on piezoelectric effect [11], are used in dynamometers and force-sensitive elements [12,13,14]. Moisture content indeed affected piezoelectric properties [15]. In our experiments, the observed trend showed that piezoelectric coefficient was invalid when the RH was over approximately 40%. It was widely believed that the effect roots from the change of bulk resistance of quartz. However, the explanation was incomplete, as the inside of quartz shielded from water vapor, and the bulk resistance could not be affected. Very few papers referred to this subject. Fortunately, the resistance RH sensor is similar to piezoelectric quartz’s in condition. Two differences must be pointed out: (1) The resistance of the RH sensor is small at 10^5^ Ω, and the quartz’s is much higher at 10^13^ Ω. Extreme high resistance between the two electrodes, which displays a strong dependence on the quartz-water interface, is essential for collecting and maintaining the induced charges; (2) In RH sensors, the change of resistance caused by water adsorption is beneficial and should be utilized. However, in piezoelectric dynamometers, the adsorption is ruinous and should be avoided. Therefore, compared with the research on the origin of effect, methods of isolation from water vapor are more significant.

Traditional solution is to cover insulation paste outside the sheets even when sealing the shells. There are some shortcomings: (1) the method is complex, and it is difficult to gelatinize uniformly; (2) As insulation paste increases the quality of dynamometers, natural frequency is reduced. Therefore, a novel strategy to eliminate the influence of water adsorption with quartz surfaces on piezoelectric dynamometers was proposed.

Superhydrophobicity [16] is a significant property of solid surface and has become a hot research topic in recent years [17]. Superhydrophobic surfaces can be acquired by electrochemical machining and fluoridation technology, which is used for self-cleaning [18], anti-drag [19], anti-corrosion [20], and frost resistance [21]. In recent years, researchers have conducted extensive papers on the corrosion resistance of metals such as copper [22], steel [23], and Mg [24]. Fluoridation technology was skillfully introduced to nonmetal-piezoelectric quartz crystals [25] to shield it from water vapor.

Above all, the influence of water adsorption with quartz surfaces on the piezoelectric effect was elucidated. First, a force-induced charge experiment revealed the dependence of RH. Then, a water-quartz model was developed to explain the origin of effect, in which the relationship of the three R’s (Roughness, RH, and Resistor) was discussed, respectively. Fluoridation to quartz sheets for shielding water vapor was presented. Finally, the interesting results not only showed that the model was reasonable and effective but also suggested a new method to change the structure, decrease the quality, and improve the properties of dynamometers.

## 2. Experiments about Piezoelectric Coefficients in Different RH Conditions

**Figure 1:**
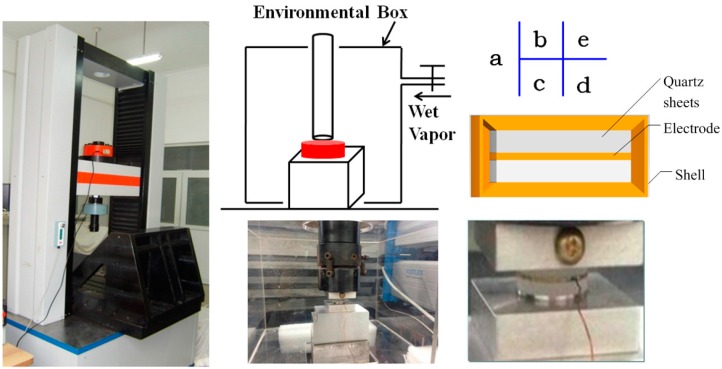
The sketch of the equipment. (**a**) Testing machines; (**b**) An environmental box sketch; (**c**) Charge amplifiers; (**d**) Force-sensitive elements; (**e**) A structure of piezoelectric dynamometers.

**Figure 2:**
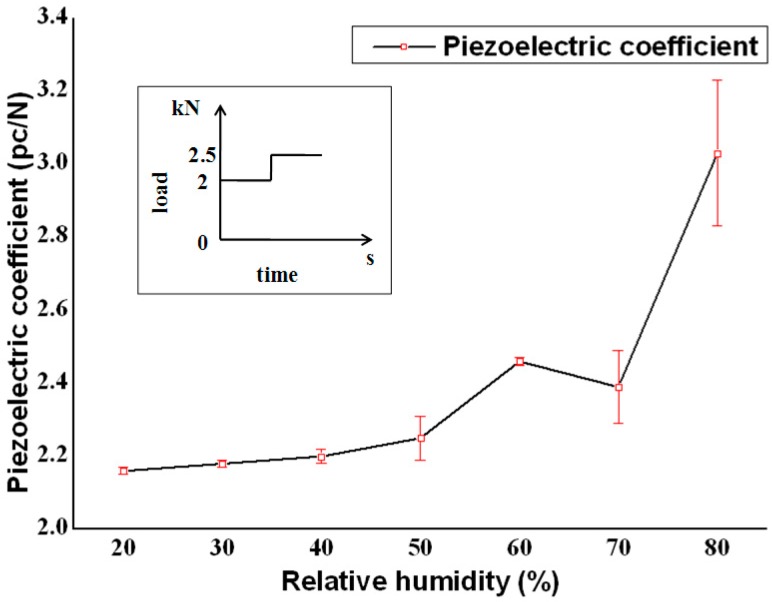
The piezoelectric coefficient dependence of RH.

Piezoelectric coefficients were measured by using the force-induced charge method in different RH conditions. A common structure of piezoelectric dynamometers is shown in Figure 1e. The element was composed of up, middle, down electrodes and two sheets of quartz, like a sandwich, with a shell. A force-sensitive element was designed, referred by standard dynamometers, as shown in Figure 1d. In order to intensify the effect of interactions of water, the thickness of the quartz sheets was increased to 3 mm, compared with the common one 1 mm. The sheets were exposed outside. Forces were loaded by using testing machines, machined by the Changchun Research Institute for Mechanical Science CO. LTD, Ji Lin, China, as shown in Figure 1a. The values of the reload force and load force were 2 kN and 0.5 kN, respectively. An environmental box, a sketch of which is shown in Figure 1b, regulated the relative humidity of the atmosphere, where wet vapor was input. Induced charges were measured by using charge amplifiers (Kistler 5018), as shown in Figure 1c. The results are shown in Figure 2. Error bars stand for measurement data for every time. The loading force curve is shown in the inset.

As the observed trend shows, piezoelectric coefficients were invalid when the RH exceeded approximately 40% because the theoretical value is 2.31 pc/N in room temperature. It was concluded that RH seriously influenced the piezoelectric property of dynamometers. The error ranges became even wider with a high RH. It was indicated that piezoelectric dynamometers could not be assembled in high RH ambient, and dynamometers could not be used without protection. It was widely believed that the effect roots from the change in the bulk resistance of the quartz. However, it was found that the explanation was incomplete, and an accurate model was developed, described in the next section.

## 3. A Water-Quartz Model

**Figure 3:**
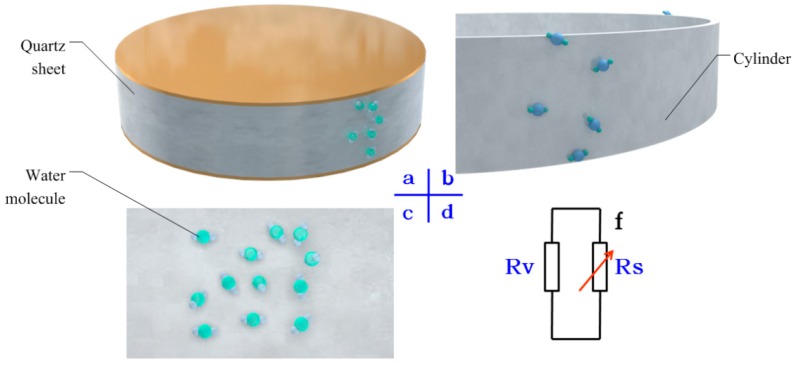
Piezoelectric dynamometers and interactions of water. (**a**) Sheets and water molecules; (**b**) Cylinder and water molecules; (**c**)Flat surface and water molecules; (**d**) The sheet resistance and bulk resistance.

A water-quartz model about the effect of water adsorption at quartz was developed and is described here. As loaded, equal positive and negative charges appeared on the electrodes. Assume there were massive water molecules around the crystals, as shown in Figure 3a. Up and down surfaces of sheets were not influenced because of their identical potentials, but the cylinder adsorbed vapor directly, as shown in Figure 3b.The cylinder to the flat surface and the front view of the expanding sheet is shown in Figure 3c. As the RH improved, a water film appeared and became thick.

Above all, based on the above analysis, a water-quartz model is here presented. Between the positive and negative electrodes, there were two kinds of resistance: a bulk resistance and a sheet resistance, which were in parallel, as Figure 3d shows. The bulk resistance kept the same, as the inside structure of quartz was invariant in different RH conditions. The sheet resistance changed with RH variation inversely. The total resistance $R_{\text{in}}$, is composed of the two resistances, as shown in Equation (1), dependent on the RH. Therefore, the sheet resistance of the cylinder was significant for investigating the effect mechanism, and an experiment was carried out.

(1) $$\frac{1}{R_{in}}=\frac{1}{R_{v}}+\frac{1}{R_{s}}$$

## 4. Experiments about the Relationships between Roughness, RH, and Resistance of Sheets

**Figure 4:**
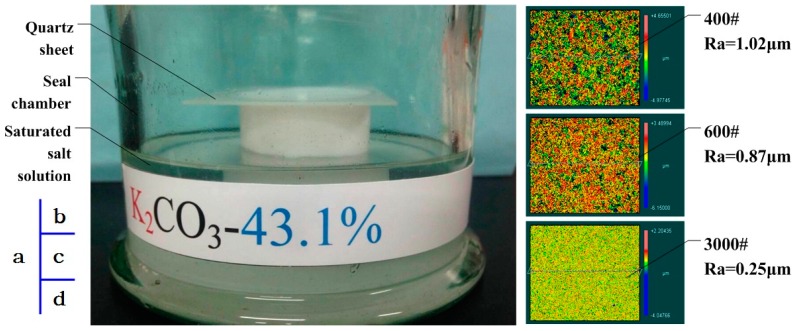
The roughness of surfaces grinded by different sand. (**a**) A seal chamber containing an appropriate saturated salt solution; (**b**) Surface topography of 400# sheets; (**c**) Surface topography of 600# sheets; (**d**) Surface topography of 3000# sheets.

**Figure 5:**
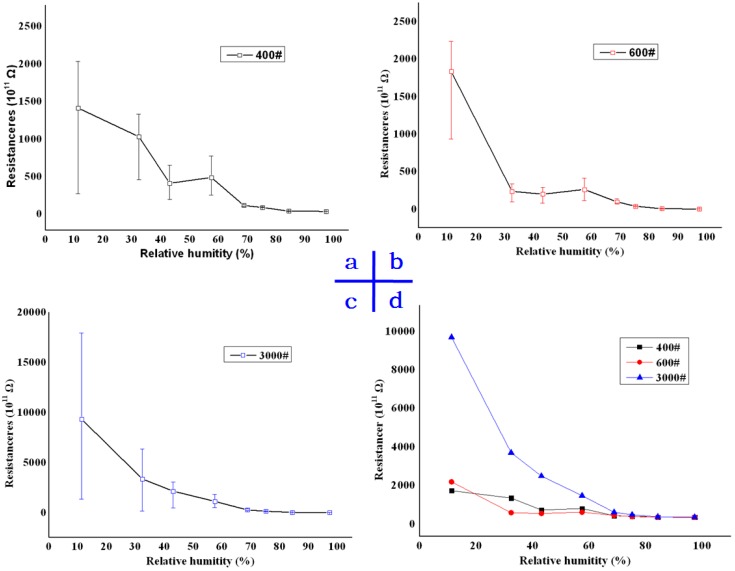
The sheet resistances with different RHs and different roughness. (**a**) 400# sheet resistance vs. relative humidity; (**b**) 600# sheet resistance vs. relative humidity; (**c**) 3000# sheet resistance vs. relative humidity; (**d**) A comparison between thethree sheets.

In order to research resistance variation of the cylinder with different RHs, plane sheets were prepared and made equivalent to the extended surface of the cylinder, as Figure 3c shows. The sheet resistance was measured using Meggers (Keithley 6517B and 8009). Limited by Keithley 8009, quartz sheets were machined with the size 70 × 70 × 1 mm, x-direction. By keeping the test samples in a seal chamber containing an appropriate saturated salt solution, the humidity of sheets was controlled. The sheet and seal chamber are shown in Figure 4a. Salts and RH are listed in Table 1. In order to investigate the effect of different roughness on water adsorption, sheets were grinded by 400#, 600# and 3000# sand. The roughness of the surfaces was measured with a ZYGO laser scanning profile-meter, and the images are shown in Figure 4b–d, obtaining a roughness of 0.25 μm, 0.87 μm, and 1.02 μm, respectively. After one hour, the sample was taken out from the chamber and measured. The results are shown in Figure 5.

## 5. Discussion

First, as Figure 5 shows, the sheet resistance of the three samples decreased while water was adsorbed onto quartz surfaces. In a low-RH environment, the sheet resistance was about 10^14^ Ω. When the RH was over 40%, the measured data was below 10^12^ Ω. According to Equation (1), the total resistance of dynamometers was below 10^12^ Ω. When a charge amplifier was used for the secondary meters, the force-sensitive element was considered as a power source for charges, whose internal resistance should have been above 10^13^ Ω, possibly infinity, which mean tan open circuit relative to charge amplifiers. From 40% RH to 90% RH, the internal resistance was too low to hinder inside currents. The induced charges could not be maintained at the two electrodes and measured by charge amplifiers. This made sense of the illogical data in Figure 2.

**Figure 6:**
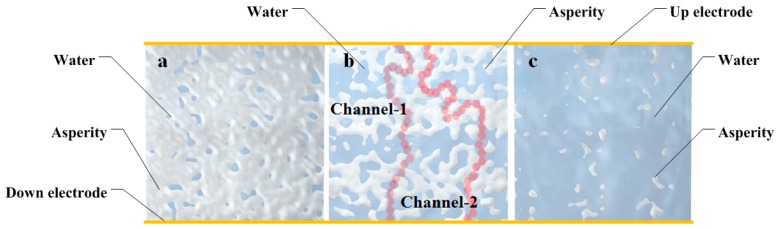
Water adsorption process on a rough surface. (**a**) Adsorption in low humidity; (**b**) Discharge channels forming; (**c**) Water film forming.

Second, water molecule adsorption proceeded while RH increased. A three-dimensional simulation diagram, as shown in Figure 6, was used to speculate the water adsorption process in rough surfaces and to explain the resistance variation.

Below 40% RH, as Figure 6a shows, very few water molecules were adsorbed at the rough surface, and most of the image is asperities of quartz. Water molecules were isolated, and the bridge between the up and down electrodes was not formed. Thus, the resistances were high. It is suggested that a new charge channel forms in up to a 40% RH environment, as the resistor declined obviously. From 40% RH to 60% RH, the dependence of sheet resistances on RH was ambiguity, and fluctuated in a narrow range, and there was no obvious decline, as shown in Figure 5a–c. It was inferred that the area of water adsorption increased and different channels appeared, as Figure 6 b shows. Those channels, such as Channel-1 and Channel-2, were extremely unstable, and a change in the environment could make them break down or reconnect. Discharge paths of induced charges were random every time. In conclusion, in the course of the 40% RH to 60% RH environments, the sheet resistances were low and unordered.

Above 70% RH, an interesting phenomenon was observed, as shown in Figure 5a–c: the variation range of the sheet resistances was small. This phenomenon illustrated that fragmented adsorption region expanded, and an integrated water film had been already formed, as Figure 6c shows. Most of the image was water. In conclusion, resistors decreased, and the variation range became narrower. Though high RH was a drawback for piezoelectric quartz, these observations drew some useful insights to high-resistance measurement.

Third, roughness was one of the most significant effects on the sheet resistances. The absolute value of resistances was affected by the roughness. Smooth surfaces obtained higher resistance than the rough ones, as shown in Figure 5d. The roughness was also connected with the formation of water films. The rough surface, which meant deep valleys and high peaks in micro-topography, made it hard for water films to form. As shown in Figure 5a,b, the resistance variation of the 400# and 600# samples is still obvious at 60% RH. However, 3000# samples were limited at the same RH, as shown in Figure 5c. Therefore, the optimization machining method of sheet circle surfaces was essential so that the sheets could obtain smooth side surfaces.

The side of piezoelectric quartz sheets was machined and was limited by several methods, such as drilling if the shape of the sheets was cylindrical or cutting by fixed abrasive diamond wire saw [26] if the shape was square. The roughness of the side was limited by the machining technologies. For example, the surface roughness of water drilling was 0.37 μm. Therefore, it was impractical to shield water vapor via the optimizing roughness of sheets. Generally, extra protection-insulation paste was used for shielding the water vapor. It had three drawbacks: (1) The operation of painting was complex and it was difficult to keep the uniformity; (2) Insulation paste decreased the stiffness of dynamometers; (3) It was more significant that the weight increased, which had a strong impact on the natural frequency of dynamometers.

In conclusion, a new method of shielding water vapor for quartz surfaces in dynamometers was presented next.

## 6. Fluoridation

Superhydrophobicity has been widely researched to change the properties of sold surfaces that are mainly used at metal material, such as Mg alloys, and nonmetal material, such as glass. The use of quartz crystals in piezoelectric dynamometers had not yet been reported. However, significant matters had been mentioned: the idea of superhydrophobicity was introduced for shielding water vapor, but not for the splash. The valuation of effectiveness, which used the experiment equipment as shown in Figure 1, was different from the metal superhydrophobicity.

Superhydrophobic surfaces can be traditionally fabricated via two strategies: (1) assemble layer-by-layer micro or nano structures inspired by lotus leaves, as air may become trapped in the spaces among nano-nipples to form an air water barrier; or (2) cover with a low surface energy film to resist with water to form a self-assembled barrier.

**Figure 7:**
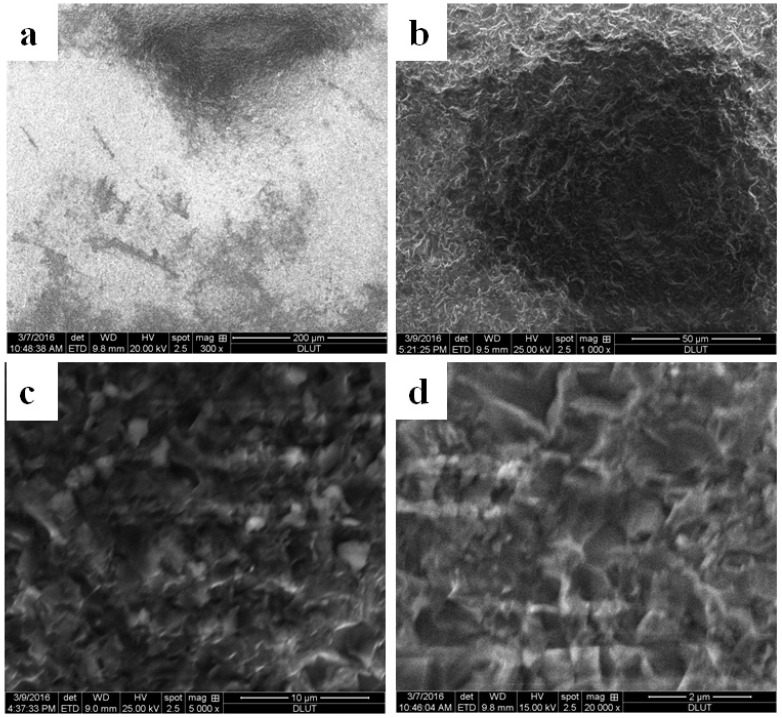
The SEM images of the quartz crystal sheet surfaces.

The surface morphologies of quartz crystal sheets were investigated using a scanning electron microscope (SEM) (EDAX, Q45, FEI Co., Hillsboro, TX, USA). The SEM images are shown in Figure 7 with different magnifications. The roughness of the sample was 0.45 μm. As Figure 7a shows, micrometer-scale protrusions and pits existed in the surfaces. Obvious grinding marks are shown in Figure 7b, and shallow pits appeared in the image at 1000× magnification. Figure 7c shows a magnified image of the shallow pits. Some asperities were shown at 5000× magnification, and more microcosmic pits were among them. There were no obvious cracks. The SEM image at 20,000× magnification is shown in Figure 7d. The structure is the same as that in Figure 7c. There was no obvious layer-by-layer micro- or nano- structure similar to the lotus leaf surface at the quartz sheets. Therefore, limited by the processing conditions, superhydrophobic surfaces of quartz in piezoelectric dynamometers were investigated by covering films.

**Figure 8:**
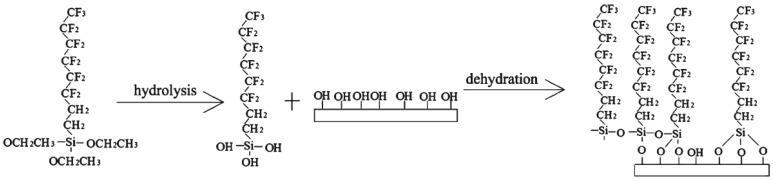
Formation mechanism of the self-assembled fluoroalkylsilane (FAS) film on the sheet surfaces.

A fluoroalkylsilane (FAS, C8F13H4Si(OC2H5)3, Degussa Co., Bitterfeld, Germany), which involved the –CF3 group with a low surface energy of 6.7 mJ/m^2^ and the –CF2 group with a surface energy of 18 mJ/m^2^, was used to reduce the free energy of the quartz sheet surfaces. The silicon ethoxide (Si-OC2H5) functional groups underwent hydrolysis and dehydration to form a self-assembled film via middle product silanols (Si-OH). The self-assembly of FAS on the quartz crystal sheet surfaces is shown in Figure 8.

**Figure 9:**
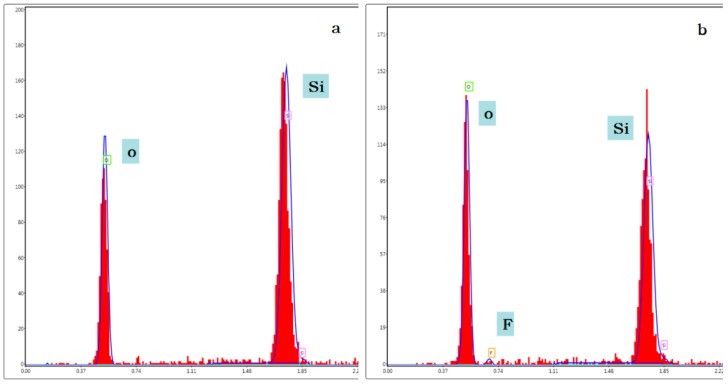
The energy-dispersive spectroscopy (EDS) spectra of the quartz crystal sheet surfaces. (**a**) Uncovered sheets; (**b**) Sheets covered with FAS film.

The sheets were covered with FAS film for an hour and then dried in 80 °C for four hours. Their chemical compositions were investigated using energy-dispersive spectroscopy (EDS) equipped with the scanning electron microscope (EDAX, Q45, FEI Co., Hillsboro, TX, USA). Figure 9a,b show the EDS spectra of the untreated and treated quartz sheet surfaces, respectively. Compared to the untreated surfaces, the surfaces were treated by FAS, which mainly consists of elements Si, O, and F, indicating that the FAS film had self-assembled on the quartz surfaces.

**Figure 10:**
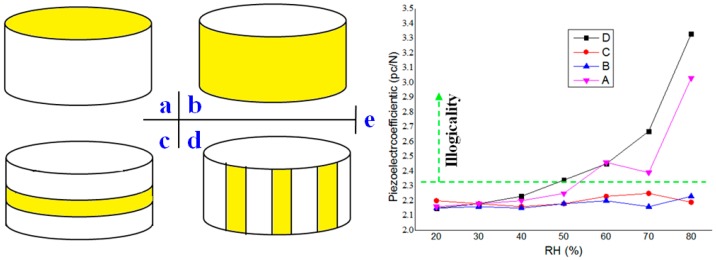
Different FAS patterns and results. (**a**) Fluoridation at up/down surface; (**b**) Fluoridation at side surface; (**c**) Ring fluoridation at side surface; (**d**)Axial fluoridation along the side surface; (**e**) Piezoelectric coefficients of different FAS patterns in different RH.

There were two kinds of surfaces: up/down planes and side surface. The effects of their wet ability on piezoelectric properties were different, as mentioned in Section 3. Therefore, fluoridation of each surface was investigated, respectively, as shown in Figure 10a,b. In order to verify the model in Part 3, different fluoridation patterns aim at the side surface were considered further. The patterns, as shown in Figure 10c,d, were to examine discharging paths of induced charges. Parties in yellow were covered with the FAS film, as shown in Figure 10a–d.

By using the equipment in Figure 1, the curves of piezoelectric coefficients toward the RH are illustrated in Figure 10e. The blue imaginary line was the theory data 2.31 pc/N. As shown in Figure 10e, Patterns A and D of FAS were invalid, as, in high RH, the coefficient exceeded the blue imaginary line. However, patterns B and C successfully protected the quartz sheets from water adsorption. The results of Patterns A and B illustrated that the side surfaces of sheets were significant for the influence of the interactions of water with quartz surfaces on the piezoelectric effect. Interestingly, the observed results of Patterns C and D clearly demonstrated that induced charges were neutralized along the side surface with the axial direction. “A tiny moat,” a FAS film, which was less than 1 mm wide and parallel with the up/down surfaces in the pattern C condition, could prevent the charges to pass successfully.

In conclusion, a novel and logical strategy-FAS film was introduced, in order to shield quartz from water vapor. The experiment results indicate that the model in Party 3 was reasonable. Moreover, the approach, proven here to be effective, can be readily applied to piezoelectric dynamometers.

## 7. Conclusions

In summary, the influence of water adsorption on quartz surfaces on piezoelectric dynamometers in humid ambience was elucidated. A new model of water-quartz adsorption was built. Invalid piezoelectric parameters in a high RH environment were caused by the water adsorption on the side surface of the quartz sheets. That made the sheet resistance of the internal resistance of force-sensitive elements decrease sharply. A new inspiration from superhydrophobic surface was first presented to deal with water molecules invading. A FAS film was first introduced to the piezoelectric crystal sheets, which possibly replaced the current sealing pattern-insulation paste and overturned the current structure of dynamometers with further research. The advantages of FAS films compared with insulation paste led to weight reduction, volume reduction, and easy operation.

In addition, based on water vapor influence, relationships between the roughness, resistance, and RH were discussed. The RH dependence of resistance suggested that a stable adsorbed water layer forms approaching to 70% RH. The water layer contributed to neutralize the induced charges. The adsorbed water layer not only decreased the resistance but also reduced the fluctuation of the high resistance, which could draw some useful insights to high resistance measurements.

## Figures and Tables

**Table 1:** The saturated salt solution and relative humidity (RH).

Salt	LiCl	MgCl_2_	K_2_CO_3_	NaBr	KI	NaCl	KCl	K_2_SO_4_
**%RH**	11.3	32.8	43.1	57.5	68.8	75.3	84.3	97.3

## References

[1] Jung I., Dikin D., Park S., Cai W., Mielke S.L., Ruoff R.S. (2008). Effect of Water Vapor on Electrical Properties of Individual Reduced Graphene Oxide Sheets. J. Phys. Chem. C.

[2] Traversa E. (1995). Ceramic sensors for humidity detection: the state-of-the-art and future developments. Sens. Actuators B Chem..

[3] Su M., Wang J., Hao Y. (2011). Development of Y^3+^ and Mg^2+^-doped zirconia thick film humidity sensors. Mater. Chem. Phys..

[4] Gao X., Yan X., Yao X., Xu L., Zhang K., Zhang J., Yang B., Jiang L. (2007). The Dry-Style Antifogging Properties of Mosquito Compound Eyes and Artificial Analogues Prepared by Soft Lithography. Adv. Mater..

[5] Xu W., Song J., Sun J., Lu Y., Yu Z. (2011). Rapid fabrication of large-area, corrosion-resistant superhydrophobic Mg alloy surfaces. ACS Appl. Mater. Interfaces.

[6] Notman R., Walsh T.R. (2009). Molecular Dynamics Studies of the Interactions of Water and Amino Acid Analogues with Quartz Surfaces. Langmuir.

[7] Bonnaud P.A., Coasne B., Pellenq R.J.M. (2010). Molecular simulation of water confined in nanoporous silica. J. Phys. Condens. Matter.

[8] Miranda P.B., Xu L., Shen Y.R., Salmeron M. (1998). Icelike Water Monolayer Adsorbed on Mica at Room Temperature. Phys. Rev. Lett..

[9] Polycarpou A.A., Etsion I. (1999). Analytical Approximations in Modeling Contacting Rough Surfaces. J. Tribol. Trans. ASME.

[10] Wolfer P., Lehmann A., Schaffner G. (2009). Measuring Sensor Comprising a Pre-Stressing Device. U.S. Patent.

[11] Meitzler A.H., Tiersten H.F., Warner A.W., Berlincourt D., Coquin G.A., Welsh F.S. (1988). IEEE Standard on Piezoelectricity.

[12] Li Y.J., Zhang J., Jia Z.Y., Qian M. (2009). A novel piezoelectric 6-component heavy force/moment sensor for huge heavy-load manipulator’s gripper. Mech. Syst. Signal Process..

[13] Jia Z.Y., Lin S., Liu W. (2010). Measurement method of six-axis load sharing based on the Stewart platform. Measurement.

[14] Gao C.Y., Li W.Q., Sun B.Y. (2010). Research on the piezoelectric torsional effect of a rectangular quartz disc and a novel drilling dynamometer. Measurement.

[15] Reinish G.B., Nowick A.S. (1975). Piezoelectric properties of bone as functions of moisture content. Nature.

[16] Cohen N., Dotan A., Dodiuk H., Kenig S. (2015). Superhydrophobic Coatings and Their Durability. Mater. Manuf. Process..

[17] Yu F.P., Yuan D.R., Zhan X., Zhang S.J. Investigation of langanite and langatate single crystals for high temperature sensing. Proceedings of the IEEESymposium on Piezoelectricity, Acoustic Waves, and Device Applications.

[18] Cristofolini L., Marchetti A., Cappello A., Viceconti M. (2000). A novel transducer for the measurement of cement-prosthesis interface forces in cemented orthopaedic devices. Med. Eng. Phys..

[19] Rosochowski A. (2001). Technical feasibility of a three-axis force transducer for measuring pressure and friction on the model die surface-prototype development. J. Mater. Process. Technol..

[20] Zhang S., Zheng Y., Kong H., Xin J., Frantz E., Shrout T.R. (2009). Characterization of high temperature piezoelectric crystals with an ordered langasite structure. J. Appl. Phys..

[21] Stefanescu D.M. (2011). Handbook of Force Transducers: Principles and Components.

[22] Rao A.V., Latthe S.S., Mahadik S.A., Kappenstein C. (2011). Mechanically stable and corrosion resistant superhydrophobic sol-gel coatings on copper substrate. Appl. Surf. Sci..

[23] Wu B., Zhou M., Li J., Ye X., Li G., Cai L. (2009). Superhydrophobic surfaces fabricated by microstructuring of stainless steel using a femtosecond laser. Appl. Surf. Sci..

[24] She Z., Li Q., Wang Z., Li L., Chen F., Zhou J. (2013). Researching the fabrication of anticorrosion superhydrophobic surface on magnesium alloy and its mechanical stability and durability. Chem. Eng. J..

[25] Esmeryan K., Radeva E., Avramov I. (2015). Durable superhydrophobic carbon soot coatings for sensor applications. J. Phys. D Appl. Phys..

[26] Jin L., Wang F., Zhao L. (2014). Ping-pong fixed abrasive diamond wire saw slicing piezoelectric crystal quartz. Mater. Phys. Mech..

